# High proportion of TAFRO syndrome in Thai adult Castleman’s disease patients: a 10-year experience

**DOI:** 10.1007/s00277-018-3269-x

**Published:** 2018-02-20

**Authors:** Weerapat Owattanapanich, Wikanda Pholmoo, Tawatchai Pongpruttipan, Noppadol Siritanaratkul

**Affiliations:** 1 Division of Hematology, Department of Medicine, Faculty of Medicine Siriraj Hospital, Mahidol University, Bangkok, Thailand; 2 Department of Pathology, Faculty of Medicine Siriraj Hospital, Mahidol University, Bangkok, Thailand

**Keywords:** Castleman’s disease, TAFRO syndrome, Lymphadenopathy, Pleural effusion, Ascites

## Abstract

Castleman’s disease (CD) is a rare lymphoproliferative disorder, and its prevalence in Thailand is not known. This 10-year period study investigated the prevalence of CD in Thailand, and the clinical characteristics and outcomes of Thai CD patients, with special focus on the existence and prevalence of TAFRO syndrome. TAFRO syndrome is defined as CD with thrombocytopenia, anasarca, fever, reticulin fibrosis, and organomegaly. Thirty-three CD patients diagnosed and treated at Siriraj Hospital during January 2007 to December 2016 were included. The prevalence of CD was 1.4 per 1,000,000 patients/10 years. Median age was 46 years, with slight female predominance. Six patients were assigned to the TAFRO group. A high proportion of TAFRO syndrome (18.2%) was found among Thai adult CD patients. In addition to routine TAFRO diagnostic criteria, significantly lower hemoglobin and albumin levels were observed in the TAFRO group than in the non-TAFRO group. Treatment outcomes of CD patients were complete remission (52%), stable disease (30%), and death (13%). Three-year overall survival in the non-TAFRO group and TAFRO group was 88 and 50%, respectively. While most CD patients had a good prognosis, severe cases with TAFRO syndrome had poor outcome.

## Introduction

Castleman’s disease (CD) or angiofollicular lymph node hyperplasia is a rare lymphoproliferative disorder [1]. A 2014 study from the USA reported an estimated 10-year prevalence of 2.4 cases per one million population [2]. CD involves an abnormal proliferation of cells of the lymphatic system that is similar in many ways to lymphomas, but it is a slowly progressive disease [1]. The pathogenesis of the disease is not known, and diagnosis is based on histopathologic features [3, 4]. Affected lymph nodes are histopathologically classified as hyaline-vascular type (HV), plasma cell variant (PC), or hyaline-vascular-plasma cell type (mixed type) [3]. CD is also clinicopathologically classified as either unicentric CD (UCD) or multicentric CD (MCD) [1]. CD patients usually have no obvious clinical manifestations, with diagnosis usually being made at annual checkup or after investigating mild abnormal symptoms, such as lymphadenopathy and fever. Patient symptoms are characterized by systemic manifestations of inflammation and B cell hyperactivity, such as generalized lymphadenopathies, fever, night sweating, weight loss, and multiple organ involvement, which can resemble autoimmune disease [3, 4]. MCD associated with human herpes virus-8 (HHV-8) or human immunodeficiency virus (HIV) infection that developed in patients with severe inflammation due to overproduction of interleukin-6 (IL-6) was reported in Western countries [3, 5].

In 2012, TAFRO syndrome was defined as CD with thrombocytopenia, anasarca, fever, reticulin fibrosis, and organomegaly [4]. TAFRO syndrome is a systemic inflammatory disorder that occurs in patients with no existing autoimmune diseases. Patients can present with clinical manifestations of bone marrow, pleura, peritoneum, kidney, liver, and lymph node involvement [4, 6]. The definite cause of TAFRO syndrome is currently unknown [4].

Two prior studies in the histopathologic features of CD have been reported from Thailand. A 1997 study reported on 12 cases of CD in Thai patients [7], and a 2002 case report profiled a Thai patient with coexisting CD and Kaposi’s sarcoma [8]. However, the prevalence of CD and TAFRO syndrome in Thai population is unknown. Accordingly, this study set forth to investigate the 10-year prevalence of CD in Thailand, and the clinical characteristics and outcomes of Thai CD patients, with special focus on the existence and prevalence of TAFRO syndrome.

## Design and methods

**Fig. 1:**
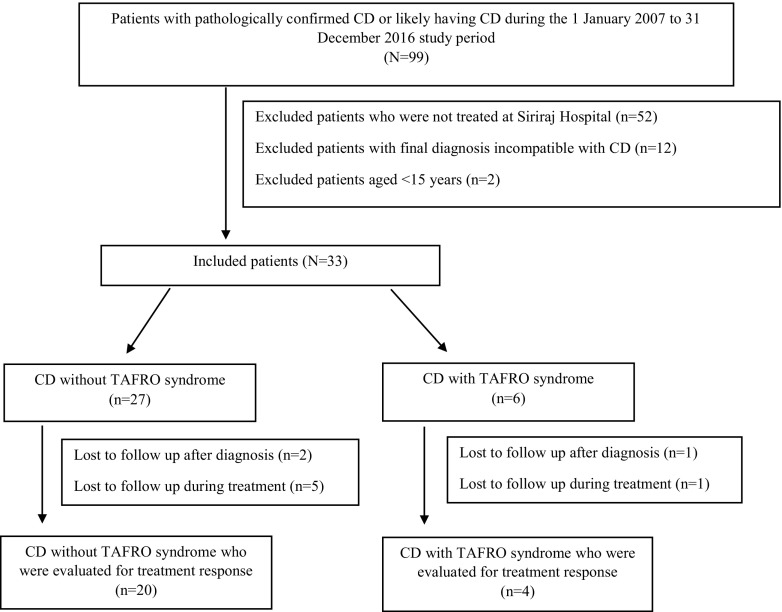
Flow diagram of patient recruitment

The 10-year period study was conducted in Thai patients aged greater than 15 years that were diagnosed (pathologically confirmed) and treated at Siriraj Hospital during the January 1, 2007, to December 31, 2016, study period. Siriraj Hospital is the Thailand’s largest national tertiary referral center. The protocol for this study involved a pathology-based reconfirmation of histopathologic reports compatible with CD, which was followed by a hospital-based chart review to analyze for prevalence, clinical characteristics, and clinical outcome in patients diagnosed with and treated for CD. Severe cases that satisfied the criteria for diagnosis of TAFRO syndrome were also identified. Patients were excluded if repeated review of pathologic tissues failed to conclusively establish a diagnosis of CD, if patients were aged less than 15 years, or if patients had insufficient clinical data (Fig. 1). According to the 2009 criteria for diagnosis of TAFRO syndrome, a patient must have at least 3 of 5 of the following factors: thrombocytopenia, anasarca, fever, reticulin fibrosis, and/or organomegaly [4]. The protocol for this study was approved by the Siriraj Institutional Review Board (SIRB), Faculty of Medicine Siriraj Hospital, Mahidol University, Bangkok, Thailand (COA no. Si 062/2016).

### Statistical analysis

All data analyses were performed using SPSS Statistics for Windows version 18 (SPSS, Inc., Chicago, IL, USA). Clinical characteristics are presented as median and interquartile range, continuous variables as mean ± standard deviation, and categorical variables as frequency and percentage. Prevalence of CD and TAFRO syndrome are shown as percentage. Continuous data were compared between groups using Student’s *t* test or Mann-Whitney U test. Categorical data were compared between groups using chi-square test or Fisher’s exact test. Binary logistic regression analyses were used to determine the relationships between variables, with an odds ratio (OR) and 95% confidence interval (CI) being calculated for each significant variable. The results of log rank test and Kaplan-Meier survival analysis were compared between Thai CD patients with and without TAFRO syndrome. A *p* value less than 0.05 was regarded as being statistically significant.

## Results

### The prevalence of CD

A total of 33 cases of CD were identified from 24,082,401 cases that were treated at Siriraj Hospital during the 10-year study period. Our review of pathologic records revealed 99 patients with history of histopathologic diagnosis of CD at our center. Sixty-six of those patients were excluded, as follows: no clinical record and/or were not treated at our hospital (52 patients); re-review of pathology reports revealed findings incompatible with CD (12 patients); and aged less than 15 years (two patients). The remaining 33 cases were included in our exploration and analysis of clinical manifestations and outcomes. The prevalence rate of CD in this study was 0.00014% per 10 years (1.4 per 1,000,000 patients per 10 years; 95% confidence interval (CI) 0.94–1.92).

### Clinical characteristics and outcomes of CD patients

Mean age of 33 Thai adult CD patients was 45.7 years (standard deviation 18.6). Gender proportion was 57.6 and 42.4% for women and men, respectively. One-third of patients sought treatment due to lymph node enlargement. Other common clinical manifestations consisted of B symptoms in 42.4% of patients, with 24.2% having weight loss, 27.3% having fever, 6.1% having night sweating, and 15.2% having fatigue. Of the patients who had lymphadenopathy, a majority had generalized lymphadenopathy (72.7%). Just over a quarter of patients had organomegaly, including hepatosplenomegaly (18.2%), hepatomegaly (3%), and splenomegaly (6.1%). The only cardiovascular finding was pericardial effusion in 9.1% of patients. Respiratory symptoms, such as pleural effusion, occurred in 21.2% of cases—all without pleuritic chest pain. Ascites and peritonitis were found in 27.3 and 3% of patients, respectively. Twenty-two percent of patients had proteinuria, but overt renal dysfunction, defined as creatinine clearance less than 60 ml/min/1.73m^2^, was observed in 27.6% of patients. Twelve percent of patients had bone marrow involvement. Overall, 31% of CD patients had multiple organ involvement at first diagnosis.

Most initial laboratory, renal function, liver function, and complete blood count test findings were in normal range, except mean globulin level, which was slightly increased (4.54 g/dl). Nine of 33 patients underwent antinuclear antibody (ANA) testing, with two cases being found positive. Approximately 10% of patients (2/23 patients) were positive for HIV. HHV-8 and EBV tests were performed in four and one cases, respectively, with all five of those cases having a negative result. Histopathologic analysis revealed hyaline-vascular type CD in 18 of 33 cases (54.5%). Plasma cell variant and mixed type was found in 6.1 and 24.2% of cases, respectively. MCD and UCD was found in 75.8 and 24.2% of cases, respectively (Table 1).

**Table 1:** Immunology, serology, histopathology, centricity, and treatment outcomes in 33 Thai Castleman’s disease patients

Serology testing	*n*	Positive (%)	Negative (%)	Not performed (%)
HHV-8	4	0.0	12.1	87.9
HIV	23	6.1 (*n* = 2)	63.6	30.3
EBV	1	0.0	3.2	96.8
Histopathology type	*n*	Valid percentage (%)		
HV type	18	54.5		
PC type	2	6.1		
Mixed type	8	24.2		
Type not identified	5	15.2		
Centricity	*n*	Valid percentage (%)		
Unicentricity	8	24.2		
Multicentricity	25	75.8		
Treatment	*n*	Valid percentage (%)		
Treatment	16	53.3		
Chemotherapy	7	23.3		
-Steroid alone/steroid-consisted regimen	7	23.3		
-Rituximab-consisted regimen	2	6.7		
-CHOP regimen	1	3.3		
-CVP regimen	3	10.0		
Surgery	11	36.7		
Watchful waiting	12	40.0		
Response	*n*	Valid percentage (%)		
Complete remission	12	52.2		
Partial remission	1	4.3		
Stable disease	7	30.4		
Death	3	13.0		
Loss to follow-up	14	58.3		
Follow-up duration (months), median (range)		8.77 (0.07 to 97.41)		
Three-year overall survival (%)		82.0		
Castleman’s disease	*n* = 33	100%		
TAFRO syndrome (2009)	6	18.2		
Non-TAFRO syndrome (2009)	27	81.8		
TAFRO syndrome (2012)	5	15.2		
Non-TAFRO syndrome (2012)	28	84.8		

Forty percent of patients were closely followed, with no immediate need for treatment. However, 36.7% of patients underwent total excision for diagnosis and treatment. A fifth of surgical patients required chemotherapy or immunosuppressive treatment, including steroid alone/steroid-consisted regimen (23.3%), CVP regimen (10%), rituximab-consisted regimen (6.7%), and CHOP regimen (3.3%). Treatment outcomes included complete remission, stable disease, and death in 52.2, 30.4, and 13% of patients, respectively.

### Prevalence and characteristics of TAFRO patients

We identified 6 of 33 patients (18.2%) that satisfied the criteria for TAFRO syndrome, which translates to a prevalence of TAFRO syndrome of 0.000025% per 10 years (0.25 per 1,000,000 patients per 10 years; 95% CI 0.09–0.54).

The demographic and clinical characteristics of TAFRO patients are shown in Table 2. Mean age of TAFRO patients was 44.0 ± 16.3 years, with male predominance (83.3%). All TAFRO syndrome patients had multiple lymphadenopathies, with 66.7 and 80% of cases having intrathoracic and intra-abdominal lymph nodes, respectively. A comparison of baseline characteristics between the TAFRO and non-TAFRO groups revealed a significantly higher rate of pericardial effusion in the TAFRO group (*p* = 0.004). Similarly, rates of B symptoms, pleural effusion, ascites, anasarca, gastrointestinal involvement, proteinuria, and multiple organ involvement were significantly higher in the TAFRO group than in the non-TAFRO group (Table 2).

**Table 2:** Demographic and clinical characteristics compared between Thai Castleman’s disease patients with and without TAFRO syndrome

	CD without TAFRO syndrome (*n* = 27)	CD with TAFRO syndrome (*n* = 6)	*p* value	OR (95% CI)
	Positive results (%)	Positive results (%)		
Gender			0.062	
Male	33.3%	83.3%		
Female	66.7%	16.7%		
Age (years), mean ± SD	46. ± 19.4	44 ± 16.3	0.813	
Clinical presentation	59.2%	100%	0.077	
Palpable mass (lymph node)	40.7%	0.0%		
Edema	7.4%	0.0%		
Fever	0.0%	66.7%		
Fatigue	3.7%	33.3%		
B symptoms	29.6%	100%	**0.003**	N/A
Weight loss	29.6%	0.0%	0.296	
Fever	11.1%	100%	***<*** **0.001**	N/A
Night sweating	7.4%	0.0%	1	
Fatigue	7.4%	50.0%	**0.031**	12.5 (1.45–107.63)
Lymphadenopathy	100%	100%	–	
Superficial LN	51.9%	50.0%	1	
Intrathoracic LN	55.6%	66.7%	1	
Intra-abdominal LN	6.9%	80.0%	0.628	
Single LN	33.3%	0.0%	0.156	
Multiple LN	66.7%	100%	0.156	
Organomegaly	15.0%	100%	0.838	
Hepatomegaly	0.0%	16.7%		
Splenomegaly	7.4%	0.0%		
Hepatosplenomegaly	3.7%	83.3%		
Multiple organ involvement	15.4%	100%	***<*** **0.001**	N/A
Cardiovascular system	7.4%	50.0%	**0.031**	12.5 (1.45–107.63)
Cardiomegaly	7.4%	16.7%	0.464	
Pericardial effusion	0.0%	50.0%	**0.004**	N/A
Respiratory	30.0%	50.0%	0.06	
Pleural effusion	7.4%	83.3%	**< 0.001**	62.5 (4.71–829.3)
Pleuritis	0.0%	0.0%	–	
Gastrointestinal system	22.2%	100%	**0.001**	N/A
Ascites	11.1%	100%	**< 0.001**	N/A
Peritonitis	0.0%	16.7%	0.182	
Renal system	29.6%	83.3%	**0.025**	11.88 (1.19–118.5)
Proteinuria	7.7%	83.3%	**0.001**	60 (4.52–797.1)
Renal dysfunction	25.0%	40.0%	0.597	
Anasarca	14.8%	100%	**0.001**	N/A
Bone marrow involvement	16.7%	0.0%	0.559	
Myelofibrosis	0.0%	0.0%	–	
Stage			**< 0.001**	N/A
Stage I	29.6%	0.0%		
Stage II	44.4%	0.0%		
Stage III	18.5%	0.0%		
Stage IV	7.4%	100%		

A *p* value < 0.05 indicates statistical significance

*CD* Castleman’s disease, *CI* confidence interval, *SD* standard deviation, *LN* lymph node, *N/A* not applicable

Laboratory results showed a significant difference for mean hemoglobin between the TAFRO and non-TAFRO groups (9.2 vs. 11.7 g/dl, respectively; *p* = 0.025), and for mean albumin between groups (2.7 vs. 3.6 g/dl, respectively; *p* = 0.047). Other laboratory findings were not significantly different between groups (Table 3). Serologic testing revealed that 20% of TAFRO syndrome patients (1/5 patients) were positive for HIV. The most common histopathologic subtype in TAFRO syndrome patients was mixed type (50%).

**Table 3:** Age, laboratory, immunology, serology, histopathology, centricity, and treatment outcomes compared between Thai Castleman’s disease patients with and without TAFRO syndrome

	CD without TAFRO syndrome					CD with TAFRO syndrome					*p* value
	*n*	Mean ± SD	Median	25th/75th percentile	Min-max	*n*	Mean ± SD	Median	25th/75th percentile	Min-max	
Age (years)	27	46 ± 19.4				6	44 ± 16.3				0.813
Hb (g/dl)	27	11.7 ± 2.4				6	9.2 ± 1.7				**0.025**
Plt (x10^3^/mcl)	27	294.2 ± 143.4				6		117	93/259	46–363	0.095
Alb (mg/l)	23	3.6 ± 0.9				6	2.7 ± 1				**0.047**
LDH (U/l)	16	280.9 ± 128.5				5	401.6 ± 122.1				0.058
BUN (mg/dl)	24		11	9.8/16.8	7.9–38	6		16.6	11.7/33	9–51.2	0.153
Cr (mg/dl)	25	1.0 ± 0.4				6	1.0 ± 0.3				0.564
GFR (ml/min/1.73m^2^)	23		81.8	47/100	23–250	6	69.9 ± 25.7				0.418
			CD without TAFRO syndrome				CD with TAFRO syndrome				*p* value
			Positive (%)				Positive (%)				
Response											**0.016**
CR			(12/20) 60%				(0/4) 0%				
PR			(0/20) 0%				(1/4) 25%				
SD			(5/20) 25%				(1/4) 25%				
Death			(3/20) 13%				(2/4) 50%				
Three-year overall survival (%)			88%				50%				0.125

A *p* value < 0.05 indicates statistical significance

*CD* Castleman’s disease, *SD* standard deviation, *Hb* hemoglobin, *Plt,* platelet, *Alb* albumin, *LDH* lactate dehydrogenase, *BUN* blood urea nitrogen, *Cr* creatinine, *GFR* glomerular filtration rate, *CR* complete response, *PR* partial response, *SD* stable disease

There were nine patients lost to follow-up after diagnosis or during treatment (seven patients in the non-TAFRO group and two patients in the TAFRO group). Therefore, a total of 24 patients (20 non-TAFRO and 4 TAFRO) were evaluated for treatment response (Fig. 1). Twenty percent of patients (5/25 patients) in the non-TAFRO group and 40% of patients (2/5 patients) in the TAFRO group required chemotherapy and immunosuppressive agent (*p* = 0.565). Treatment of TAFRO syndrome included rituximab-based regimen (20%), CHOP regimen (20%), and CVP regimen (20%). Treatment outcomes were partial response, stable disease, and death in 25, 25, and 50% of patients, respectively. No patient had complete response. Three-year overall survival in the non-TAFRO group and TAFRO group was 88 and 50%, respectively (*p* = 0.125).

## Discussion

TAFRO syndrome was first described in 2009 [4], and revised diagnostic criteria for this disorder were proposed in 2012 and 2015 [4, 9]. While many studies in CD and TAFRO syndrome have been published, this is the first study to describe the prevalence, clinical characteristics, and outcomes of adult CD patients in Thailand. Moreover, 6 of the 33 patients that we identified with CD also satisfied the 2009 diagnostic criteria for TAFRO syndrome.

The prevalence of CD in this study was 1.4 per 1,000,000 patients per 10 years. Median age of CD patients was 46 years. A prior Western systematic analysis found a median age of 33 and 38 years for females and males, respectively [10]. In the present study, CD was more common in women than men (57.6 vs. 42.4%), which is similar to prior study [4]. Nearly half of our patients sought treatment due to lymph node enlargement. Most CD patients in this study had no hepatosplenomegaly, while all 6 TAFRO syndrome patients had hepatomegaly and/or splenomegaly. Multiple organ involvement was found in 31.3% of CD patients, to include pericardial effusion, cardiomegaly, pleural effusion, ascites, peritonitis, proteinuria, and renal dysfunction. Regarding bone marrow involvement, 16.7% of CD patients were found to be affected at first diagnosis.

In this study, immunologic testing was not frequently performed. Serologic testing, especially anti-HIV testing, was performed in 23 cases. Two (8.7%) of those patients tested positive for HIV, which was similar to the 7.7% rate reported in a 2011 Western study [10]. Histopathologic analysis found hyaline-vascular type to be the most common subtype (64.3%), which was consistent with previous study (58%) [10]. In contrast, centricity results between this study and previous study varied widely. UCD was found in 24.2% of cases in this study, as compared to the 73.7% rate reported by Talat and Schulte [10].

Regarding treatment, 40% of patients did not require immediate treatment, but they were closely followed. Twenty-three percent of patients required chemotherapy or immunosuppressive treatment that included steroid alone/steroid-consisted regimen, CVP regimen, rituximab, and/or CHOP regimen, which was similar to the treatment regimens prescribed in previous studies [11–14]. The treatment outcomes were complete remission, stable disease, and death in about 52, 30, and 13% of cases, respectively, which was similar to the 15% mortality rate reported in a previous study [10].

Studies from Japan defined CD with severe symptoms as TAFRO syndrome in 2009 and 2012 [4, 14]. Six of 33 CD patients in this study satisfied the 2009 diagnostic criteria for TAFRO syndrome. Interestingly, the proportion of TAFRO syndrome in CD patients in the present study was higher than the proportion in a previous study [18.2% (6/33) patients vs. 0.7% (2/273) patients, respectively] [15]. The data permits us to postulate that Asian population are more prone to developing severe CD (TAFRO syndrome) than Western population [1, 4–6, 9, 12–14]. The median age of CD with TAFRO syndrome was 44 years with male predominance, which was different from a Japanese study that found a median age of 56 years with female predominance [4]. All patients with CD and TAFRO syndrome in this study had B symptoms, multiple lymphadenopathies, anasarca, and multiple organ involvement. Notably—in addition to meeting the diagnostic criteria for TAFRO syndrome, significantly lower hemoglobin and lower albumin levels were shown in the TAFRO group than in the non-TAFRO group in this study. We prefer to use the 2009 criteria for TAFRO diagnosis, because it has higher sensitivity for detecting TAFRO cases than the 2012 and 2015 criteria. Furthermore, the 2012 and 2015 criteria failed to detect some severe cases of CD that ended up having a poor treatment outcome.

Treatment regimens of TAFRO syndrome patients in this study included chemotherapy and immunosuppressive agent (40%), chemotherapy and immunosuppressive agent containing rituximab-consisted regimen (20%), CHOP regimen (20%), and CVP regimen (20%). Treatment outcomes were partial response (25%), stable disease (25%), and death (50%). No TAFRO patient had complete response. In this study, the prognosis of TAFRO syndrome patients was clearly worse than the prognosis of CD patients.

This study has some mentionable limitations. First, due to the rarity of this disorder, the size of our study population was relatively small. As a result, our study may have lacked sufficient power to identify all significant associations. Second, our center is Thailand’s largest tertiary referral hospital, which means that we are often referred patients with complicated and intransigent conditions. As such, it is possible that the prevalence rate of CD in this study might be higher than the prevalence rate in general population.

## Conclusion

The prevalence of CD in Thailand is very low; however, a high proportion of TAFRO syndrome was found in Thai adult CD patients compared to a previous study. Although most CD patients had a good prognosis, severe cases with TAFRO syndrome had poor outcome.
